# Supplementary material for: Dynalign II: common secondary structure prediction for RNA homologs with domain insertions
Source: Nucleic Acids Res. 2014 Nov 21;42(22):13939–48. doi: 10.1093/nar/gku1172 (PMC4267632; doi:10.1093/nar/gku1172)
Supplement: SUPPLEMENTARY DATA [file supp_gku1172_nar-02021-z-2014-File012.zip › manual/javadoc/RNAstructure_java_interface/source/startup/RNAstructureBackendCalculatorProxyJNI.html]

RNAstructureBackendCalculatorProxyJNI


JavaScript is disabled on your browser.


- Overview
- Package
- Class
- Tree
- Index
- Help

- Prev Class
- Next Class

- Frames
- No Frames

- All Classes

- Summary:
- Nested |
- Field |
- Constr |
- Method

- Detail:
- Field |
- Constr |
- Method


RNAstructure\_java\_interface.source.startup

## Class RNAstructureBackendCalculatorProxyJNI

- java.lang.Object
- - RNAstructure\_java\_interface.source.startup.RNAstructureBackendCalculatorProxyJNI

- ---

    

  ```
   class RNAstructureBackendCalculatorProxyJNI
  extends Object
  ```

- - ### Constructor Summary

    Constructors

    Constructor and Description

    `RNAstructureBackendCalculatorProxyJNI()`
  - ### Method Summary

    Methods

    | Modifier and Type | Method and Description |
    | --- | --- |
    | `static void` | `delete_RNAstructureBackendCalculator(long jarg1)` |
    | `static long` | `new_RNAstructureBackendCalculator()` |
    | `static void` | `RNAstructureBackendCalculator_activateMultilign(long jarg1, RNAstructureBackendCalculator jarg1_)` |
    | `static void` | `RNAstructureBackendCalculator_activateTurboFold(long jarg1, RNAstructureBackendCalculator jarg1_)` |
    | `static void` | `RNAstructureBackendCalculator_addMultilignTuple(long jarg1, RNAstructureBackendCalculator jarg1_, String jarg2, String jarg3)` |
    | `static void` | `RNAstructureBackendCalculator_addTurboFoldTuple(long jarg1, RNAstructureBackendCalculator jarg1_, String jarg2, String jarg3)` |
    | `static String` | `RNAstructureBackendCalculator_buildAllSubDataStructure(long jarg1, RNAstructureBackendCalculator jarg1_, String jarg2, boolean jarg3)` |
    | `static String` | `RNAstructureBackendCalculator_buildBifoldDataStructure(long jarg1, RNAstructureBackendCalculator jarg1_, String jarg2, String jarg3, boolean jarg4)` |
    | `static String` | `RNAstructureBackendCalculator_buildBipartitionDataStructure(long jarg1, RNAstructureBackendCalculator jarg1_, String jarg2, String jarg3, boolean jarg4)` |
    | `static String` | `RNAstructureBackendCalculator_buildDynalignDataStructure(long jarg1, RNAstructureBackendCalculator jarg1_, String jarg2, String jarg3, boolean jarg4)` |
    | `static String` | `RNAstructureBackendCalculator_buildEfn2DataStructure(long jarg1, RNAstructureBackendCalculator jarg1_, String jarg2, boolean jarg3)` |
    | `static String` | `RNAstructureBackendCalculator_buildFoldDataStructure(long jarg1, RNAstructureBackendCalculator jarg1_, String jarg2, boolean jarg3)` |
    | `static String` | `RNAstructureBackendCalculator_buildMaxExpectDataStructure(long jarg1, RNAstructureBackendCalculator jarg1_, String jarg2)` |
    | `static String` | `RNAstructureBackendCalculator_buildOligoScreenDataStructure(long jarg1, RNAstructureBackendCalculator jarg1_)` |
    | `static String` | `RNAstructureBackendCalculator_buildOligoWalkDataStructure(long jarg1, RNAstructureBackendCalculator jarg1_, String jarg2, boolean jarg3)` |
    | `static String` | `RNAstructureBackendCalculator_buildPartitionDataStructure(long jarg1, RNAstructureBackendCalculator jarg1_, String jarg2, boolean jarg3)` |
    | `static String` | `RNAstructureBackendCalculator_buildProbKnotDataStructure(long jarg1, RNAstructureBackendCalculator jarg1_, String jarg2)` |
    | `static String` | `RNAstructureBackendCalculator_buildRefoldDynalignDataStructure(long jarg1, RNAstructureBackendCalculator jarg1_, String jarg2)` |
    | `static String` | `RNAstructureBackendCalculator_buildRefoldSingleDataStructure(long jarg1, RNAstructureBackendCalculator jarg1_, String jarg2)` |
    | `static String` | `RNAstructureBackendCalculator_buildRemovePseudoknotsDataStructure(long jarg1, RNAstructureBackendCalculator jarg1_, String jarg2, boolean jarg3)` |
    | `static String` | `RNAstructureBackendCalculator_buildStochasticDataStructure(long jarg1, RNAstructureBackendCalculator jarg1_, String jarg2)` |
    | `static boolean` | `RNAstructureBackendCalculator_canFoldOligoOligo(long jarg1, RNAstructureBackendCalculator jarg1_, int jarg2)` |
    | `static boolean` | `RNAstructureBackendCalculator_canFoldOligoSelf(long jarg1, RNAstructureBackendCalculator jarg1_, int jarg2)` |
    | `static void` | `RNAstructureBackendCalculator_clearDynalignAlignmentConstraints(long jarg1, RNAstructureBackendCalculator jarg1_)` |
    | `static void` | `RNAstructureBackendCalculator_clearFoldingConstraints(long jarg1, RNAstructureBackendCalculator jarg1_, int jarg2)` |
    | `static void` | `RNAstructureBackendCalculator_deleteMultilignTuple(long jarg1, RNAstructureBackendCalculator jarg1_, long jarg2)` |
    | `static void` | `RNAstructureBackendCalculator_deleteTurboFoldTuple(long jarg1, RNAstructureBackendCalculator jarg1_, long jarg2)` |
    | `static int` | `RNAstructureBackendCalculator_determineOligoMaximum(long jarg1, RNAstructureBackendCalculator jarg1_, int jarg2)` |
    | `static void` | `RNAstructureBackendCalculator_foldOligo(long jarg1, RNAstructureBackendCalculator jarg1_, String jarg2, int jarg3, boolean jarg4, boolean jarg5, String jarg6)` |
    | `static String` | `RNAstructureBackendCalculator_getAllOligoData(long jarg1, RNAstructureBackendCalculator jarg1_, int jarg2)` |
    | `static String` | `RNAstructureBackendCalculator_getDisplayedOligo(long jarg1, RNAstructureBackendCalculator jarg1_, int jarg2)` |
    | `static String` | `RNAstructureBackendCalculator_getDynalignAlignmentConstraints(long jarg1, RNAstructureBackendCalculator jarg1_)` |
    | `static int` | `RNAstructureBackendCalculator_getDynalignAlignmentWindowSize(long jarg1, RNAstructureBackendCalculator jarg1_)` |
    | `static int` | `RNAstructureBackendCalculator_getDynalignStructureWindowSize(long jarg1, RNAstructureBackendCalculator jarg1_)` |
    | `static String` | `RNAstructureBackendCalculator_getFoldingConstraints(long jarg1, RNAstructureBackendCalculator jarg1_, int jarg2)` |
    | `static int` | `RNAstructureBackendCalculator_getFoldWindowSize(long jarg1, RNAstructureBackendCalculator jarg1_)` |
    | `static int` | `RNAstructureBackendCalculator_getGraphRegionBegin(long jarg1, RNAstructureBackendCalculator jarg1_)` |
    | `static int` | `RNAstructureBackendCalculator_getGraphRegionEnd(long jarg1, RNAstructureBackendCalculator jarg1_)` |
    | `static int` | `RNAstructureBackendCalculator_getMaxConstraintIndex(long jarg1, RNAstructureBackendCalculator jarg1_, int jarg2)` |
    | `static int` | `RNAstructureBackendCalculator_getMaxLoop(long jarg1, RNAstructureBackendCalculator jarg1_)` |
    | `static int` | `RNAstructureBackendCalculator_getMaxPair(long jarg1, RNAstructureBackendCalculator jarg1_)` |
    | `static int` | `RNAstructureBackendCalculator_getMostStableOligo(long jarg1, RNAstructureBackendCalculator jarg1_)` |
    | `static String` | `RNAstructureBackendCalculator_getMultilignCT(long jarg1, RNAstructureBackendCalculator jarg1_, int jarg2)` |
    | `static int` | `RNAstructureBackendCalculator_getMultilignMaxPairs(long jarg1, RNAstructureBackendCalculator jarg1_)` |
    | `static String` | `RNAstructureBackendCalculator_getMultilignSequenceSetData(long jarg1, RNAstructureBackendCalculator jarg1_)` |
    | `static int` | `RNAstructureBackendCalculator_getNumMultilignSequences(long jarg1, RNAstructureBackendCalculator jarg1_)` |
    | `static int` | `RNAstructureBackendCalculator_getNumTurboFoldSequences(long jarg1, RNAstructureBackendCalculator jarg1_)` |
    | `static String` | `RNAstructureBackendCalculator_getOligoLabelData(long jarg1, RNAstructureBackendCalculator jarg1_, int jarg2)` |
    | `static int` | `RNAstructureBackendCalculator_getOligoTargetLength(long jarg1, RNAstructureBackendCalculator jarg1_)` |
    | `static String` | `RNAstructureBackendCalculator_getOligoTargetSequence(long jarg1, RNAstructureBackendCalculator jarg1_)` |
    | `static int` | `RNAstructureBackendCalculator_getProgressNumber(long jarg1, RNAstructureBackendCalculator jarg1_)` |
    | `static int` | `RNAstructureBackendCalculator_getRefoldWindowSize(long jarg1, RNAstructureBackendCalculator jarg1_)` |
    | `static String` | `RNAstructureBackendCalculator_getSequenceComment(long jarg1, RNAstructureBackendCalculator jarg1_)` |
    | `static String` | `RNAstructureBackendCalculator_getSequenceData(long jarg1, RNAstructureBackendCalculator jarg1_)` |
    | `static String` | `RNAstructureBackendCalculator_getSequenceTitle(long jarg1, RNAstructureBackendCalculator jarg1_)` |
    | `static String` | `RNAstructureBackendCalculator_getStructureType(long jarg1, RNAstructureBackendCalculator jarg1_)` |
    | `static double` | `RNAstructureBackendCalculator_getSuboptimalAbsoluteDiff(long jarg1, RNAstructureBackendCalculator jarg1_)` |
    | `static float` | `RNAstructureBackendCalculator_getSuboptimalPercentDiff(long jarg1, RNAstructureBackendCalculator jarg1_)` |
    | `static double` | `RNAstructureBackendCalculator_getTemperature(long jarg1, RNAstructureBackendCalculator jarg1_)` |
    | `static String` | `RNAstructureBackendCalculator_getTurboFoldCT(long jarg1, RNAstructureBackendCalculator jarg1_, int jarg2)` |
    | `static String` | `RNAstructureBackendCalculator_getTurboFoldSaveFile(long jarg1, RNAstructureBackendCalculator jarg1_, int jarg2)` |
    | `static String` | `RNAstructureBackendCalculator_getTurboFoldSequenceSetData(long jarg1, RNAstructureBackendCalculator jarg1_)` |
    | `static String` | `RNAstructureBackendCalculator_readDynalignAlignmentConstraintsFile(long jarg1, RNAstructureBackendCalculator jarg1_, String jarg2)` |
    | `static String` | `RNAstructureBackendCalculator_readFoldingConstraintsFile(long jarg1, RNAstructureBackendCalculator jarg1_, String jarg2, int jarg3)` |
    | `static void` | `RNAstructureBackendCalculator_readSequenceData(long jarg1, RNAstructureBackendCalculator jarg1_, String jarg2)` |
    | `static String` | `RNAstructureBackendCalculator_runAllSub(long jarg1, RNAstructureBackendCalculator jarg1_, String jarg2, float jarg3, double jarg4)` |
    | `static String` | `RNAstructureBackendCalculator_runBifold(long jarg1, RNAstructureBackendCalculator jarg1_, String jarg2, float jarg3, int jarg4, int jarg5, boolean jarg6, boolean jarg7)` |
    | `static String` | `RNAstructureBackendCalculator_runBipartition(long jarg1, RNAstructureBackendCalculator jarg1_, String jarg2)` |
    | `static String` | `RNAstructureBackendCalculator_runDynalign(long jarg1, RNAstructureBackendCalculator jarg1_, String jarg2, String jarg3, String jarg4, String jarg5, double jarg6, int jarg7, int jarg8, int jarg9, float jarg10, boolean jarg11)` |
    | `static String` | `RNAstructureBackendCalculator_runDynalignRefold(long jarg1, RNAstructureBackendCalculator jarg1_, String jarg2, String jarg3, String jarg4, int jarg5, int jarg6, int jarg7, int jarg8)` |
    | `static String` | `RNAstructureBackendCalculator_runEfn2(long jarg1, RNAstructureBackendCalculator jarg1_, String jarg2, boolean jarg3)` |
    | `static String` | `RNAstructureBackendCalculator_runFold(long jarg1, RNAstructureBackendCalculator jarg1_, String jarg2, int jarg3, int jarg4, int jarg5, boolean jarg6)` |
    | `static String` | `RNAstructureBackendCalculator_runMaxExpect(long jarg1, RNAstructureBackendCalculator jarg1_, String jarg2, double jarg3, int jarg4, int jarg5, double jarg6)` |
    | `static String` | `RNAstructureBackendCalculator_runMultilign(long jarg1, RNAstructureBackendCalculator jarg1_, int jarg2, int jarg3, int jarg4, int jarg5, double jarg6, boolean jarg7, double jarg8, int jarg9, int jarg10, String jarg11, boolean jarg12, boolean jarg13)` |
    | `static String` | `RNAstructureBackendCalculator_runOligoScreen(long jarg1, RNAstructureBackendCalculator jarg1_, String jarg2, String jarg3, boolean jarg4)` |
    | `static String` | `RNAstructureBackendCalculator_runOligoWalk(long jarg1, RNAstructureBackendCalculator jarg1_, String jarg2, int jarg3, String jarg4, boolean jarg5, int jarg6, int jarg7, String jarg8, int jarg9, int jarg10)` |
    | `static String` | `RNAstructureBackendCalculator_runPartition(long jarg1, RNAstructureBackendCalculator jarg1_, String jarg2)` |
    | `static String` | `RNAstructureBackendCalculator_runPseudoknotPrediction(long jarg1, RNAstructureBackendCalculator jarg1_, String jarg2, int jarg3, int jarg4)` |
    | `static String` | `RNAstructureBackendCalculator_runPseudoknotRemoval(long jarg1, RNAstructureBackendCalculator jarg1_, String jarg2, boolean jarg3)` |
    | `static String` | `RNAstructureBackendCalculator_runRefold(long jarg1, RNAstructureBackendCalculator jarg1_, String jarg2, int jarg3, int jarg4, int jarg5)` |
    | `static String` | `RNAstructureBackendCalculator_runStochastic(long jarg1, RNAstructureBackendCalculator jarg1_, String jarg2, int jarg3, int jarg4)` |
    | `static String` | `RNAstructureBackendCalculator_runTurboFoldMaximumExpectedAccuracy(long jarg1, RNAstructureBackendCalculator jarg1_, double jarg2, int jarg3, double jarg4, int jarg5, int jarg6, double jarg7)` |
    | `static String` | `RNAstructureBackendCalculator_runTurboFoldPseudoknot(long jarg1, RNAstructureBackendCalculator jarg1_, double jarg2, int jarg3, int jarg4, int jarg5)` |
    | `static String` | `RNAstructureBackendCalculator_runTurboFoldThreshold(long jarg1, RNAstructureBackendCalculator jarg1_, double jarg2, int jarg3, double jarg4)` |
    | `static String` | `RNAstructureBackendCalculator_setCleavedNucleotide(long jarg1, RNAstructureBackendCalculator jarg1_, int jarg2, int jarg3)` |
    | `static String` | `RNAstructureBackendCalculator_setDoubleStrandedNucleotide(long jarg1, RNAstructureBackendCalculator jarg1_, int jarg2, int jarg3)` |
    | `static String` | `RNAstructureBackendCalculator_setDynalignAlignmentConstraint(long jarg1, RNAstructureBackendCalculator jarg1_, int jarg2, int jarg3)` |
    | `static String` | `RNAstructureBackendCalculator_setForcedHelix(long jarg1, RNAstructureBackendCalculator jarg1_, int jarg2, int jarg3, int jarg4, int jarg5)` |
    | `static void` | `RNAstructureBackendCalculator_setMaxLoop(long jarg1, RNAstructureBackendCalculator jarg1_, int jarg2)` |
    | `static void` | `RNAstructureBackendCalculator_setMaxPair(long jarg1, RNAstructureBackendCalculator jarg1_, int jarg2)` |
    | `static String` | `RNAstructureBackendCalculator_setModifiedNucleotide(long jarg1, RNAstructureBackendCalculator jarg1_, int jarg2, int jarg3)` |
    | `static String` | `RNAstructureBackendCalculator_setProhibitedHelix(long jarg1, RNAstructureBackendCalculator jarg1_, int jarg2, int jarg3, int jarg4, int jarg5)` |
    | `static void` | `RNAstructureBackendCalculator_setSequenceComment(long jarg1, RNAstructureBackendCalculator jarg1_, String jarg2)` |
    | `static void` | `RNAstructureBackendCalculator_setSequenceData(long jarg1, RNAstructureBackendCalculator jarg1_, String jarg2)` |
    | `static void` | `RNAstructureBackendCalculator_setSequenceTitle(long jarg1, RNAstructureBackendCalculator jarg1_, String jarg2)` |
    | `static void` | `RNAstructureBackendCalculator_setSHAPEFile(long jarg1, RNAstructureBackendCalculator jarg1_, String jarg2)` |
    | `static void` | `RNAstructureBackendCalculator_setSHAPEParam1(long jarg1, RNAstructureBackendCalculator jarg1_, double jarg2)` |
    | `static void` | `RNAstructureBackendCalculator_setSHAPEParam2(long jarg1, RNAstructureBackendCalculator jarg1_, double jarg2)` |
    | `static void` | `RNAstructureBackendCalculator_setSHAPEType(long jarg1, RNAstructureBackendCalculator jarg1_, boolean jarg2)` |
    | `static String` | `RNAstructureBackendCalculator_setSingleStrandedNucleotide(long jarg1, RNAstructureBackendCalculator jarg1_, int jarg2, int jarg3)` |
    | `static void` | `RNAstructureBackendCalculator_setTemperature(long jarg1, RNAstructureBackendCalculator jarg1_, double jarg2)` |
    | `static void` | `RNAstructureBackendCalculator_writeDynalignAlignmentConstraintsFile(long jarg1, RNAstructureBackendCalculator jarg1_, String jarg2)` |
    | `static String` | `RNAstructureBackendCalculator_writeFastaFile(long jarg1, RNAstructureBackendCalculator jarg1_, String jarg2)` |
    | `static String` | `RNAstructureBackendCalculator_writeFoldingConstraintsFile(long jarg1, RNAstructureBackendCalculator jarg1_, String jarg2, int jarg3)` |
    | `static String` | `RNAstructureBackendCalculator_writeSequenceFile(long jarg1, RNAstructureBackendCalculator jarg1_, String jarg2)` |

    - ### Methods inherited from class java.lang.Object

      `clone, equals, finalize, getClass, hashCode, notify, notifyAll, toString, wait, wait, wait`

- - ### Constructor Detail


    - #### RNAstructureBackendCalculatorProxyJNI

      ```
      RNAstructureBackendCalculatorProxyJNI()
      ```
  - ### Method Detail


    - #### delete\_RNAstructureBackendCalculator

      ```
      public static final void delete_RNAstructureBackendCalculator(long jarg1)
      ```


    - #### new\_RNAstructureBackendCalculator

      ```
      public static final long new_RNAstructureBackendCalculator()
      ```


    - #### RNAstructureBackendCalculator\_activateMultilign

      ```
      public static final void RNAstructureBackendCalculator_activateMultilign(long jarg1,
                                                         RNAstructureBackendCalculator jarg1_)
      ```


    - #### RNAstructureBackendCalculator\_activateTurboFold

      ```
      public static final void RNAstructureBackendCalculator_activateTurboFold(long jarg1,
                                                         RNAstructureBackendCalculator jarg1_)
      ```


    - #### RNAstructureBackendCalculator\_addMultilignTuple

      ```
      public static final void RNAstructureBackendCalculator_addMultilignTuple(long jarg1,
                                                         RNAstructureBackendCalculator jarg1_,
                                                         String jarg2,
                                                         String jarg3)
      ```


    - #### RNAstructureBackendCalculator\_addTurboFoldTuple

      ```
      public static final void RNAstructureBackendCalculator_addTurboFoldTuple(long jarg1,
                                                         RNAstructureBackendCalculator jarg1_,
                                                         String jarg2,
                                                         String jarg3)
      ```


    - #### RNAstructureBackendCalculator\_buildAllSubDataStructure

      ```
      public static final String RNAstructureBackendCalculator_buildAllSubDataStructure(long jarg1,
                                                                  RNAstructureBackendCalculator jarg1_,
                                                                  String jarg2,
                                                                  boolean jarg3)
      ```


    - #### RNAstructureBackendCalculator\_buildBifoldDataStructure

      ```
      public static final String RNAstructureBackendCalculator_buildBifoldDataStructure(long jarg1,
                                                                  RNAstructureBackendCalculator jarg1_,
                                                                  String jarg2,
                                                                  String jarg3,
                                                                  boolean jarg4)
      ```


    - #### RNAstructureBackendCalculator\_buildBipartitionDataStructure

      ```
      public static final String RNAstructureBackendCalculator_buildBipartitionDataStructure(long jarg1,
                                                                       RNAstructureBackendCalculator jarg1_,
                                                                       String jarg2,
                                                                       String jarg3,
                                                                       boolean jarg4)
      ```


    - #### RNAstructureBackendCalculator\_buildDynalignDataStructure

      ```
      public static final String RNAstructureBackendCalculator_buildDynalignDataStructure(long jarg1,
                                                                    RNAstructureBackendCalculator jarg1_,
                                                                    String jarg2,
                                                                    String jarg3,
                                                                    boolean jarg4)
      ```


    - #### RNAstructureBackendCalculator\_buildEfn2DataStructure

      ```
      public static final String RNAstructureBackendCalculator_buildEfn2DataStructure(long jarg1,
                                                                RNAstructureBackendCalculator jarg1_,
                                                                String jarg2,
                                                                boolean jarg3)
      ```


    - #### RNAstructureBackendCalculator\_buildFoldDataStructure

      ```
      public static final String RNAstructureBackendCalculator_buildFoldDataStructure(long jarg1,
                                                                RNAstructureBackendCalculator jarg1_,
                                                                String jarg2,
                                                                boolean jarg3)
      ```


    - #### RNAstructureBackendCalculator\_buildMaxExpectDataStructure

      ```
      public static final String RNAstructureBackendCalculator_buildMaxExpectDataStructure(long jarg1,
                                                                     RNAstructureBackendCalculator jarg1_,
                                                                     String jarg2)
      ```


    - #### RNAstructureBackendCalculator\_buildOligoScreenDataStructure

      ```
      public static final String RNAstructureBackendCalculator_buildOligoScreenDataStructure(long jarg1,
                                                                       RNAstructureBackendCalculator jarg1_)
      ```


    - #### RNAstructureBackendCalculator\_buildOligoWalkDataStructure

      ```
      public static final String RNAstructureBackendCalculator_buildOligoWalkDataStructure(long jarg1,
                                                                     RNAstructureBackendCalculator jarg1_,
                                                                     String jarg2,
                                                                     boolean jarg3)
      ```


    - #### RNAstructureBackendCalculator\_buildPartitionDataStructure

      ```
      public static final String RNAstructureBackendCalculator_buildPartitionDataStructure(long jarg1,
                                                                     RNAstructureBackendCalculator jarg1_,
                                                                     String jarg2,
                                                                     boolean jarg3)
      ```


    - #### RNAstructureBackendCalculator\_buildProbKnotDataStructure

      ```
      public static final String RNAstructureBackendCalculator_buildProbKnotDataStructure(long jarg1,
                                                                    RNAstructureBackendCalculator jarg1_,
                                                                    String jarg2)
      ```


    - #### RNAstructureBackendCalculator\_buildRefoldDynalignDataStructure

      ```
      public static final String RNAstructureBackendCalculator_buildRefoldDynalignDataStructure(long jarg1,
                                                                          RNAstructureBackendCalculator jarg1_,
                                                                          String jarg2)
      ```


    - #### RNAstructureBackendCalculator\_buildRefoldSingleDataStructure

      ```
      public static final String RNAstructureBackendCalculator_buildRefoldSingleDataStructure(long jarg1,
                                                                        RNAstructureBackendCalculator jarg1_,
                                                                        String jarg2)
      ```


    - #### RNAstructureBackendCalculator\_buildRemovePseudoknotsDataStructure

      ```
      public static final String RNAstructureBackendCalculator_buildRemovePseudoknotsDataStructure(long jarg1,
                                                                             RNAstructureBackendCalculator jarg1_,
                                                                             String jarg2,
                                                                             boolean jarg3)
      ```


    - #### RNAstructureBackendCalculator\_buildStochasticDataStructure

      ```
      public static final String RNAstructureBackendCalculator_buildStochasticDataStructure(long jarg1,
                                                                      RNAstructureBackendCalculator jarg1_,
                                                                      String jarg2)
      ```


    - #### RNAstructureBackendCalculator\_canFoldOligoOligo

      ```
      public static final boolean RNAstructureBackendCalculator_canFoldOligoOligo(long jarg1,
                                                            RNAstructureBackendCalculator jarg1_,
                                                            int jarg2)
      ```


    - #### RNAstructureBackendCalculator\_canFoldOligoSelf

      ```
      public static final boolean RNAstructureBackendCalculator_canFoldOligoSelf(long jarg1,
                                                           RNAstructureBackendCalculator jarg1_,
                                                           int jarg2)
      ```


    - #### RNAstructureBackendCalculator\_clearDynalignAlignmentConstraints

      ```
      public static final void RNAstructureBackendCalculator_clearDynalignAlignmentConstraints(long jarg1,
                                                                         RNAstructureBackendCalculator jarg1_)
      ```


    - #### RNAstructureBackendCalculator\_clearFoldingConstraints

      ```
      public static final void RNAstructureBackendCalculator_clearFoldingConstraints(long jarg1,
                                                               RNAstructureBackendCalculator jarg1_,
                                                               int jarg2)
      ```


    - #### RNAstructureBackendCalculator\_deleteMultilignTuple

      ```
      public static final void RNAstructureBackendCalculator_deleteMultilignTuple(long jarg1,
                                                            RNAstructureBackendCalculator jarg1_,
                                                            long jarg2)
      ```


    - #### RNAstructureBackendCalculator\_deleteTurboFoldTuple

      ```
      public static final void RNAstructureBackendCalculator_deleteTurboFoldTuple(long jarg1,
                                                            RNAstructureBackendCalculator jarg1_,
                                                            long jarg2)
      ```


    - #### RNAstructureBackendCalculator\_determineOligoMaximum

      ```
      public static final int RNAstructureBackendCalculator_determineOligoMaximum(long jarg1,
                                                            RNAstructureBackendCalculator jarg1_,
                                                            int jarg2)
      ```


    - #### RNAstructureBackendCalculator\_foldOligo

      ```
      public static final void RNAstructureBackendCalculator_foldOligo(long jarg1,
                                                 RNAstructureBackendCalculator jarg1_,
                                                 String jarg2,
                                                 int jarg3,
                                                 boolean jarg4,
                                                 boolean jarg5,
                                                 String jarg6)
      ```


    - #### RNAstructureBackendCalculator\_getAllOligoData

      ```
      public static final String RNAstructureBackendCalculator_getAllOligoData(long jarg1,
                                                         RNAstructureBackendCalculator jarg1_,
                                                         int jarg2)
      ```


    - #### RNAstructureBackendCalculator\_getDisplayedOligo

      ```
      public static final String RNAstructureBackendCalculator_getDisplayedOligo(long jarg1,
                                                           RNAstructureBackendCalculator jarg1_,
                                                           int jarg2)
      ```


    - #### RNAstructureBackendCalculator\_getDynalignAlignmentConstraints

      ```
      public static final String RNAstructureBackendCalculator_getDynalignAlignmentConstraints(long jarg1,
                                                                         RNAstructureBackendCalculator jarg1_)
      ```


    - #### RNAstructureBackendCalculator\_getDynalignAlignmentWindowSize

      ```
      public static final int RNAstructureBackendCalculator_getDynalignAlignmentWindowSize(long jarg1,
                                                                     RNAstructureBackendCalculator jarg1_)
      ```


    - #### RNAstructureBackendCalculator\_getDynalignStructureWindowSize

      ```
      public static final int RNAstructureBackendCalculator_getDynalignStructureWindowSize(long jarg1,
                                                                     RNAstructureBackendCalculator jarg1_)
      ```


    - #### RNAstructureBackendCalculator\_getFoldingConstraints

      ```
      public static final String RNAstructureBackendCalculator_getFoldingConstraints(long jarg1,
                                                               RNAstructureBackendCalculator jarg1_,
                                                               int jarg2)
      ```


    - #### RNAstructureBackendCalculator\_getFoldWindowSize

      ```
      public static final int RNAstructureBackendCalculator_getFoldWindowSize(long jarg1,
                                                        RNAstructureBackendCalculator jarg1_)
      ```


    - #### RNAstructureBackendCalculator\_getGraphRegionBegin

      ```
      public static final int RNAstructureBackendCalculator_getGraphRegionBegin(long jarg1,
                                                          RNAstructureBackendCalculator jarg1_)
      ```


    - #### RNAstructureBackendCalculator\_getGraphRegionEnd

      ```
      public static final int RNAstructureBackendCalculator_getGraphRegionEnd(long jarg1,
                                                        RNAstructureBackendCalculator jarg1_)
      ```


    - #### RNAstructureBackendCalculator\_getMaxConstraintIndex

      ```
      public static final int RNAstructureBackendCalculator_getMaxConstraintIndex(long jarg1,
                                                            RNAstructureBackendCalculator jarg1_,
                                                            int jarg2)
      ```


    - #### RNAstructureBackendCalculator\_getMaxLoop

      ```
      public static final int RNAstructureBackendCalculator_getMaxLoop(long jarg1,
                                                 RNAstructureBackendCalculator jarg1_)
      ```


    - #### RNAstructureBackendCalculator\_getMaxPair

      ```
      public static final int RNAstructureBackendCalculator_getMaxPair(long jarg1,
                                                 RNAstructureBackendCalculator jarg1_)
      ```


    - #### RNAstructureBackendCalculator\_getMostStableOligo

      ```
      public static final int RNAstructureBackendCalculator_getMostStableOligo(long jarg1,
                                                         RNAstructureBackendCalculator jarg1_)
      ```


    - #### RNAstructureBackendCalculator\_getMultilignCT

      ```
      public static final String RNAstructureBackendCalculator_getMultilignCT(long jarg1,
                                                        RNAstructureBackendCalculator jarg1_,
                                                        int jarg2)
      ```


    - #### RNAstructureBackendCalculator\_getMultilignMaxPairs

      ```
      public static final int RNAstructureBackendCalculator_getMultilignMaxPairs(long jarg1,
                                                           RNAstructureBackendCalculator jarg1_)
      ```


    - #### RNAstructureBackendCalculator\_getMultilignSequenceSetData

      ```
      public static final String RNAstructureBackendCalculator_getMultilignSequenceSetData(long jarg1,
                                                                     RNAstructureBackendCalculator jarg1_)
      ```


    - #### RNAstructureBackendCalculator\_getNumMultilignSequences

      ```
      public static final int RNAstructureBackendCalculator_getNumMultilignSequences(long jarg1,
                                                               RNAstructureBackendCalculator jarg1_)
      ```


    - #### RNAstructureBackendCalculator\_getNumTurboFoldSequences

      ```
      public static final int RNAstructureBackendCalculator_getNumTurboFoldSequences(long jarg1,
                                                               RNAstructureBackendCalculator jarg1_)
      ```


    - #### RNAstructureBackendCalculator\_getOligoLabelData

      ```
      public static final String RNAstructureBackendCalculator_getOligoLabelData(long jarg1,
                                                           RNAstructureBackendCalculator jarg1_,
                                                           int jarg2)
      ```


    - #### RNAstructureBackendCalculator\_getOligoTargetLength

      ```
      public static final int RNAstructureBackendCalculator_getOligoTargetLength(long jarg1,
                                                           RNAstructureBackendCalculator jarg1_)
      ```


    - #### RNAstructureBackendCalculator\_getOligoTargetSequence

      ```
      public static final String RNAstructureBackendCalculator_getOligoTargetSequence(long jarg1,
                                                                RNAstructureBackendCalculator jarg1_)
      ```


    - #### RNAstructureBackendCalculator\_getProgressNumber

      ```
      public static final int RNAstructureBackendCalculator_getProgressNumber(long jarg1,
                                                        RNAstructureBackendCalculator jarg1_)
      ```


    - #### RNAstructureBackendCalculator\_getRefoldWindowSize

      ```
      public static final int RNAstructureBackendCalculator_getRefoldWindowSize(long jarg1,
                                                          RNAstructureBackendCalculator jarg1_)
      ```


    - #### RNAstructureBackendCalculator\_getSequenceComment

      ```
      public static final String RNAstructureBackendCalculator_getSequenceComment(long jarg1,
                                                            RNAstructureBackendCalculator jarg1_)
      ```


    - #### RNAstructureBackendCalculator\_getSequenceData

      ```
      public static final String RNAstructureBackendCalculator_getSequenceData(long jarg1,
                                                         RNAstructureBackendCalculator jarg1_)
      ```


    - #### RNAstructureBackendCalculator\_getSequenceTitle

      ```
      public static final String RNAstructureBackendCalculator_getSequenceTitle(long jarg1,
                                                          RNAstructureBackendCalculator jarg1_)
      ```


    - #### RNAstructureBackendCalculator\_getStructureType

      ```
      public static final String RNAstructureBackendCalculator_getStructureType(long jarg1,
                                                          RNAstructureBackendCalculator jarg1_)
      ```


    - #### RNAstructureBackendCalculator\_getSuboptimalAbsoluteDiff

      ```
      public static final double RNAstructureBackendCalculator_getSuboptimalAbsoluteDiff(long jarg1,
                                                                   RNAstructureBackendCalculator jarg1_)
      ```


    - #### RNAstructureBackendCalculator\_getSuboptimalPercentDiff

      ```
      public static final float RNAstructureBackendCalculator_getSuboptimalPercentDiff(long jarg1,
                                                                 RNAstructureBackendCalculator jarg1_)
      ```


    - #### RNAstructureBackendCalculator\_getTemperature

      ```
      public static final double RNAstructureBackendCalculator_getTemperature(long jarg1,
                                                        RNAstructureBackendCalculator jarg1_)
      ```


    - #### RNAstructureBackendCalculator\_getTurboFoldCT

      ```
      public static final String RNAstructureBackendCalculator_getTurboFoldCT(long jarg1,
                                                        RNAstructureBackendCalculator jarg1_,
                                                        int jarg2)
      ```


    - #### RNAstructureBackendCalculator\_getTurboFoldSaveFile

      ```
      public static final String RNAstructureBackendCalculator_getTurboFoldSaveFile(long jarg1,
                                                              RNAstructureBackendCalculator jarg1_,
                                                              int jarg2)
      ```


    - #### RNAstructureBackendCalculator\_getTurboFoldSequenceSetData

      ```
      public static final String RNAstructureBackendCalculator_getTurboFoldSequenceSetData(long jarg1,
                                                                     RNAstructureBackendCalculator jarg1_)
      ```


    - #### RNAstructureBackendCalculator\_readDynalignAlignmentConstraintsFile

      ```
      public static final String RNAstructureBackendCalculator_readDynalignAlignmentConstraintsFile(long jarg1,
                                                                              RNAstructureBackendCalculator jarg1_,
                                                                              String jarg2)
      ```


    - #### RNAstructureBackendCalculator\_readFoldingConstraintsFile

      ```
      public static final String RNAstructureBackendCalculator_readFoldingConstraintsFile(long jarg1,
                                                                    RNAstructureBackendCalculator jarg1_,
                                                                    String jarg2,
                                                                    int jarg3)
      ```


    - #### RNAstructureBackendCalculator\_readSequenceData

      ```
      public static final void RNAstructureBackendCalculator_readSequenceData(long jarg1,
                                                        RNAstructureBackendCalculator jarg1_,
                                                        String jarg2)
      ```


    - #### RNAstructureBackendCalculator\_runAllSub

      ```
      public static final String RNAstructureBackendCalculator_runAllSub(long jarg1,
                                                   RNAstructureBackendCalculator jarg1_,
                                                   String jarg2,
                                                   float jarg3,
                                                   double jarg4)
      ```


    - #### RNAstructureBackendCalculator\_runBifold

      ```
      public static final String RNAstructureBackendCalculator_runBifold(long jarg1,
                                                   RNAstructureBackendCalculator jarg1_,
                                                   String jarg2,
                                                   float jarg3,
                                                   int jarg4,
                                                   int jarg5,
                                                   boolean jarg6,
                                                   boolean jarg7)
      ```


    - #### RNAstructureBackendCalculator\_runBipartition

      ```
      public static final String RNAstructureBackendCalculator_runBipartition(long jarg1,
                                                        RNAstructureBackendCalculator jarg1_,
                                                        String jarg2)
      ```


    - #### RNAstructureBackendCalculator\_runDynalign

      ```
      public static final String RNAstructureBackendCalculator_runDynalign(long jarg1,
                                                     RNAstructureBackendCalculator jarg1_,
                                                     String jarg2,
                                                     String jarg3,
                                                     String jarg4,
                                                     String jarg5,
                                                     double jarg6,
                                                     int jarg7,
                                                     int jarg8,
                                                     int jarg9,
                                                     float jarg10,
                                                     boolean jarg11)
      ```


    - #### RNAstructureBackendCalculator\_runDynalignRefold

      ```
      public static final String RNAstructureBackendCalculator_runDynalignRefold(long jarg1,
                                                           RNAstructureBackendCalculator jarg1_,
                                                           String jarg2,
                                                           String jarg3,
                                                           String jarg4,
                                                           int jarg5,
                                                           int jarg6,
                                                           int jarg7,
                                                           int jarg8)
      ```


    - #### RNAstructureBackendCalculator\_runEfn2

      ```
      public static final String RNAstructureBackendCalculator_runEfn2(long jarg1,
                                                 RNAstructureBackendCalculator jarg1_,
                                                 String jarg2,
                                                 boolean jarg3)
      ```


    - #### RNAstructureBackendCalculator\_runFold

      ```
      public static final String RNAstructureBackendCalculator_runFold(long jarg1,
                                                 RNAstructureBackendCalculator jarg1_,
                                                 String jarg2,
                                                 int jarg3,
                                                 int jarg4,
                                                 int jarg5,
                                                 boolean jarg6)
      ```


    - #### RNAstructureBackendCalculator\_runMaxExpect

      ```
      public static final String RNAstructureBackendCalculator_runMaxExpect(long jarg1,
                                                      RNAstructureBackendCalculator jarg1_,
                                                      String jarg2,
                                                      double jarg3,
                                                      int jarg4,
                                                      int jarg5,
                                                      double jarg6)
      ```


    - #### RNAstructureBackendCalculator\_runMultilign

      ```
      public static final String RNAstructureBackendCalculator_runMultilign(long jarg1,
                                                      RNAstructureBackendCalculator jarg1_,
                                                      int jarg2,
                                                      int jarg3,
                                                      int jarg4,
                                                      int jarg5,
                                                      double jarg6,
                                                      boolean jarg7,
                                                      double jarg8,
                                                      int jarg9,
                                                      int jarg10,
                                                      String jarg11,
                                                      boolean jarg12,
                                                      boolean jarg13)
      ```


    - #### RNAstructureBackendCalculator\_runOligoScreen

      ```
      public static final String RNAstructureBackendCalculator_runOligoScreen(long jarg1,
                                                        RNAstructureBackendCalculator jarg1_,
                                                        String jarg2,
                                                        String jarg3,
                                                        boolean jarg4)
      ```


    - #### RNAstructureBackendCalculator\_runOligoWalk

      ```
      public static final String RNAstructureBackendCalculator_runOligoWalk(long jarg1,
                                                      RNAstructureBackendCalculator jarg1_,
                                                      String jarg2,
                                                      int jarg3,
                                                      String jarg4,
                                                      boolean jarg5,
                                                      int jarg6,
                                                      int jarg7,
                                                      String jarg8,
                                                      int jarg9,
                                                      int jarg10)
      ```


    - #### RNAstructureBackendCalculator\_runPartition

      ```
      public static final String RNAstructureBackendCalculator_runPartition(long jarg1,
                                                      RNAstructureBackendCalculator jarg1_,
                                                      String jarg2)
      ```


    - #### RNAstructureBackendCalculator\_runPseudoknotPrediction

      ```
      public static final String RNAstructureBackendCalculator_runPseudoknotPrediction(long jarg1,
                                                                 RNAstructureBackendCalculator jarg1_,
                                                                 String jarg2,
                                                                 int jarg3,
                                                                 int jarg4)
      ```


    - #### RNAstructureBackendCalculator\_runPseudoknotRemoval

      ```
      public static final String RNAstructureBackendCalculator_runPseudoknotRemoval(long jarg1,
                                                              RNAstructureBackendCalculator jarg1_,
                                                              String jarg2,
                                                              boolean jarg3)
      ```


    - #### RNAstructureBackendCalculator\_runRefold

      ```
      public static final String RNAstructureBackendCalculator_runRefold(long jarg1,
                                                   RNAstructureBackendCalculator jarg1_,
                                                   String jarg2,
                                                   int jarg3,
                                                   int jarg4,
                                                   int jarg5)
      ```


    - #### RNAstructureBackendCalculator\_runStochastic

      ```
      public static final String RNAstructureBackendCalculator_runStochastic(long jarg1,
                                                       RNAstructureBackendCalculator jarg1_,
                                                       String jarg2,
                                                       int jarg3,
                                                       int jarg4)
      ```


    - #### RNAstructureBackendCalculator\_runTurboFoldMaximumExpectedAccuracy

      ```
      public static final String RNAstructureBackendCalculator_runTurboFoldMaximumExpectedAccuracy(long jarg1,
                                                                             RNAstructureBackendCalculator jarg1_,
                                                                             double jarg2,
                                                                             int jarg3,
                                                                             double jarg4,
                                                                             int jarg5,
                                                                             int jarg6,
                                                                             double jarg7)
      ```


    - #### RNAstructureBackendCalculator\_runTurboFoldPseudoknot

      ```
      public static final String RNAstructureBackendCalculator_runTurboFoldPseudoknot(long jarg1,
                                                                RNAstructureBackendCalculator jarg1_,
                                                                double jarg2,
                                                                int jarg3,
                                                                int jarg4,
                                                                int jarg5)
      ```


    - #### RNAstructureBackendCalculator\_runTurboFoldThreshold

      ```
      public static final String RNAstructureBackendCalculator_runTurboFoldThreshold(long jarg1,
                                                               RNAstructureBackendCalculator jarg1_,
                                                               double jarg2,
                                                               int jarg3,
                                                               double jarg4)
      ```


    - #### RNAstructureBackendCalculator\_setCleavedNucleotide

      ```
      public static final String RNAstructureBackendCalculator_setCleavedNucleotide(long jarg1,
                                                              RNAstructureBackendCalculator jarg1_,
                                                              int jarg2,
                                                              int jarg3)
      ```


    - #### RNAstructureBackendCalculator\_setDoubleStrandedNucleotide

      ```
      public static final String RNAstructureBackendCalculator_setDoubleStrandedNucleotide(long jarg1,
                                                                     RNAstructureBackendCalculator jarg1_,
                                                                     int jarg2,
                                                                     int jarg3)
      ```


    - #### RNAstructureBackendCalculator\_setDynalignAlignmentConstraint

      ```
      public static final String RNAstructureBackendCalculator_setDynalignAlignmentConstraint(long jarg1,
                                                                        RNAstructureBackendCalculator jarg1_,
                                                                        int jarg2,
                                                                        int jarg3)
      ```


    - #### RNAstructureBackendCalculator\_setForcedHelix

      ```
      public static final String RNAstructureBackendCalculator_setForcedHelix(long jarg1,
                                                        RNAstructureBackendCalculator jarg1_,
                                                        int jarg2,
                                                        int jarg3,
                                                        int jarg4,
                                                        int jarg5)
      ```


    - #### RNAstructureBackendCalculator\_setMaxLoop

      ```
      public static final void RNAstructureBackendCalculator_setMaxLoop(long jarg1,
                                                  RNAstructureBackendCalculator jarg1_,
                                                  int jarg2)
      ```


    - #### RNAstructureBackendCalculator\_setMaxPair

      ```
      public static final void RNAstructureBackendCalculator_setMaxPair(long jarg1,
                                                  RNAstructureBackendCalculator jarg1_,
                                                  int jarg2)
      ```


    - #### RNAstructureBackendCalculator\_setModifiedNucleotide

      ```
      public static final String RNAstructureBackendCalculator_setModifiedNucleotide(long jarg1,
                                                               RNAstructureBackendCalculator jarg1_,
                                                               int jarg2,
                                                               int jarg3)
      ```


    - #### RNAstructureBackendCalculator\_setProhibitedHelix

      ```
      public static final String RNAstructureBackendCalculator_setProhibitedHelix(long jarg1,
                                                            RNAstructureBackendCalculator jarg1_,
                                                            int jarg2,
                                                            int jarg3,
                                                            int jarg4,
                                                            int jarg5)
      ```


    - #### RNAstructureBackendCalculator\_setSequenceComment

      ```
      public static final void RNAstructureBackendCalculator_setSequenceComment(long jarg1,
                                                          RNAstructureBackendCalculator jarg1_,
                                                          String jarg2)
      ```


    - #### RNAstructureBackendCalculator\_setSequenceData

      ```
      public static final void RNAstructureBackendCalculator_setSequenceData(long jarg1,
                                                       RNAstructureBackendCalculator jarg1_,
                                                       String jarg2)
      ```


    - #### RNAstructureBackendCalculator\_setSequenceTitle

      ```
      public static final void RNAstructureBackendCalculator_setSequenceTitle(long jarg1,
                                                        RNAstructureBackendCalculator jarg1_,
                                                        String jarg2)
      ```


    - #### RNAstructureBackendCalculator\_setSHAPEFile

      ```
      public static final void RNAstructureBackendCalculator_setSHAPEFile(long jarg1,
                                                    RNAstructureBackendCalculator jarg1_,
                                                    String jarg2)
      ```


    - #### RNAstructureBackendCalculator\_setSHAPEParam1

      ```
      public static final void RNAstructureBackendCalculator_setSHAPEParam1(long jarg1,
                                                      RNAstructureBackendCalculator jarg1_,
                                                      double jarg2)
      ```


    - #### RNAstructureBackendCalculator\_setSHAPEParam2

      ```
      public static final void RNAstructureBackendCalculator_setSHAPEParam2(long jarg1,
                                                      RNAstructureBackendCalculator jarg1_,
                                                      double jarg2)
      ```


    - #### RNAstructureBackendCalculator\_setSHAPEType

      ```
      public static final void RNAstructureBackendCalculator_setSHAPEType(long jarg1,
                                                    RNAstructureBackendCalculator jarg1_,
                                                    boolean jarg2)
      ```


    - #### RNAstructureBackendCalculator\_setSingleStrandedNucleotide

      ```
      public static final String RNAstructureBackendCalculator_setSingleStrandedNucleotide(long jarg1,
                                                                     RNAstructureBackendCalculator jarg1_,
                                                                     int jarg2,
                                                                     int jarg3)
      ```


    - #### RNAstructureBackendCalculator\_setTemperature

      ```
      public static final void RNAstructureBackendCalculator_setTemperature(long jarg1,
                                                      RNAstructureBackendCalculator jarg1_,
                                                      double jarg2)
      ```


    - #### RNAstructureBackendCalculator\_writeDynalignAlignmentConstraintsFile

      ```
      public static final void RNAstructureBackendCalculator_writeDynalignAlignmentConstraintsFile(long jarg1,
                                                                             RNAstructureBackendCalculator jarg1_,
                                                                             String jarg2)
      ```


    - #### RNAstructureBackendCalculator\_writeFastaFile

      ```
      public static final String RNAstructureBackendCalculator_writeFastaFile(long jarg1,
                                                        RNAstructureBackendCalculator jarg1_,
                                                        String jarg2)
      ```


    - #### RNAstructureBackendCalculator\_writeFoldingConstraintsFile

      ```
      public static final String RNAstructureBackendCalculator_writeFoldingConstraintsFile(long jarg1,
                                                                     RNAstructureBackendCalculator jarg1_,
                                                                     String jarg2,
                                                                     int jarg3)
      ```


    - #### RNAstructureBackendCalculator\_writeSequenceFile

      ```
      public static final String RNAstructureBackendCalculator_writeSequenceFile(long jarg1,
                                                           RNAstructureBackendCalculator jarg1_,
                                                           String jarg2)
      ```


- Overview
- Package
- Class
- Tree
- Index
- Help

- Prev Class
- Next Class

- Frames
- No Frames

- All Classes

- Summary:
- Nested |
- Field |
- Constr |
- Method

- Detail:
- Field |
- Constr |
- Method
